# Supplementary material for: Healthy Obese Subjects Differ in Chronotype, Sleep Habits, and Adipose Tissue Fatty Acid Composition from Their Non-Healthy Counterparts
Source: Nutrients. 2020 Dec 31;13(1):119. doi: 10.3390/nu13010119 (PMC7824395; doi:10.3390/nu13010119)
Supplement: Supplementary file 1 [file nutrients-13-00119-s001.pdf]

Table S1. Energy and nutrient intake in MHO and MUO (Population 1).

Dietary Intake	MHO<br>*n* = 429	MUO<br>*n* = 429	*p*-Value
	Energy and macronutrient intake		
Energy intake (kcal)	1966.41 ± 36.73	1883.60 ± 34.74	0.103
Carbohydrates (%) [1]	41.23 ± 0.62	40.73 ± 0.59	0.570
Proteins (%) [1]	17.65 ± 0.28	17.88 ± 0.27	0.556
Lipids (%) [1]	41.96 ± 0.57	42.38 ± 0.54	0.590
SFA (%) [1]	10.42 ± 0.23	10.67 ± 0.22	0.435
MUFA (%) [1]	19.29 ± 0.35	19.03 ± 0.34	0.592
PUFA (%) [1]	4.56 ± 0.09	4.61 ± 0.09	0.732
Cholesterol (mg)	270.02 ± 11.09	278.42 ± 10.47	0.583
Fibre (g)	16.76 ± 0.53	16.05 ± 0.50	0.327
	Micronutrient intake		
Calcium (mg)	767.44 ± 22.13	721.88 ± 20.76	0.135
Iron (mg)	13.20 ± 0.28	13.15 ± 0.26	0.903
Iodine (mg)	180.18 ± 7.84	156.02 ± 7.37	**0.026**
Zinc (mg)	8.51 ± 0.23	8.50 ± 0.21	0.995
Magnesium (mg)	250.81 ± 4.74	239.90 ± 4.44	0.094
Potassium (mg)	2996.22 ± 65.05	2814.29 ± 59.83	**0.041**
Thiamine (B1) (mg)	1.46 ± 0.04	1.48 ± 0.04	0.677
Riboflavin (B2) (mg)	1.54 ± 0.05	1.46 ± 0.00	0.259
Niacin (B3) (mg)	27.75 ± 0.64	26.93 ± 0.60	0.354
Pyridoxine (B6) (mg)	1.88 ± 0.04	1.78 ± 0.04	0.06
Folic acid (B9) (mg)	208.57 ± 7.14	198.32 ± 6.69	0.297
Cobalamin (B12) (mg)	5.56 ± 0.38	5.92 ± 0.36	0.501
Vitamin C (mg)	136.03 ± 6.18	130.92 ± 5.82	0.549
Vitamin A (mg)	617.90 ± 22.65	571.24 ± 21.18	0.134
Vitamin D (mg)	7.68 ± 0.72	8.10 ± 0.69	0.678
Vitamin E (mg)	7.10 ± 0.23	7.19 ± 0.22	0.772

Data are presented as estimated means ± SEM. ANCOVA analysis was performed adjusted by Study Number, Clinical Center and energy intake. [1] Percentage of total energy intake. ANCOVA, analysis of covariance; MHO, metabolically healthy obesity; MUFA, monounsaturated fatty acids; MUO, metabolically unhealthy obesity; PUFA, polyunsaturated fatty acids; SEM, standard error of the mean; SFA, saturated fatty acids. Bold numbers highlight statistical significance.

Table S2. Hormonal concentrations (Population 2).

Variable	MHO	MUO	*p*-Value
Males	*n* = 6	*n* = 19	
17-β-Estradiol (pg/ml)	17.87±7.53	21.39±16.75	0.771
Testosterone (ng/dl)	4.40±16.75	3.37±1.49	0.092
DHEA-S (μg/dl)	127.00±105.00	168.84±173.92	0.849
SHBG (nmol/l)	48.88±28.56	29.36±18.76	0.080
Androstenedione (ng/ml)	1.68±0.66	1.53±0.91	0.588
C peptide (ng/ml)	3.46±2.31	3.53±2.33	0.915
TNF-α (pg/ml)	22.03±10.07	17.80±9.05	0.323
Leptin (ng/ml)	9.28±4.89	7.60±3.64	0.504
Pre-menopausal females	*n* = 4	*n* = 15	
17-β-Estradiol (pg/ml)	94.39±29.35	73.54±65.91	0.137
Testosterone (ng/dl)	0.13±0.06	0.33±0.32	0.234
DHEA-S (μg/dl)	101.00±46.16	134.47±99.92	0.841
SHBG (nmol/l)	63.05±44.42	61.85±52.98	0.484
Androstenedione (ng/ml)	1.09±0.20	1.80±0.99	0.079
C peptide (ng/ml)	2.70±0.78	4.87±2.68	0.089
TNF-α (pg/ml)	18.75±8.18	20.50±6.18	0.616
Leptin (ng/ml)	25.20±16.05	24.69±12.16	0.671

Post-menopausal females	$n = 9$	$n = 19$	
17-β-Estradiol (pg/ml)	11.44±2.97	16.36±23.18	0.740
Testosterone (ng/dl)	0.17±0.10	0.24±0.28	0.831
DHEA-S (μg/dl)	53.56±49.53	70.68±62.98	0.290
SHBG (nmol/l)	72.06±35.08	49.76±22.04	0.076
Androstenedione (ng/ml)	1.37±0.85	1.15±0.55	0.639
C peptide (ng/ml)	2.80±1.19	3.60±1.49	0.192
TNF-α (pg/ml)	20.56±9.61	25.63±7.06	0.192
Leptin (ng/ml)	36.77±19.62	27.46±11.77	0.248

Data are presented as mean ± SD. Comparison of hormones concentrations between MHO and MUO phenotypes were realized with Student t test or Mann Whitney U test as appropriate. DHEA-S, dehydroepiandrosterone sulfate; MHO, metabolically healthy obesity; MUO, metabolically unhealthy obesity; SD, standard deviation; SHBG, sex hormone binding globulin; TNF-α, tumor necrosis factor-α.

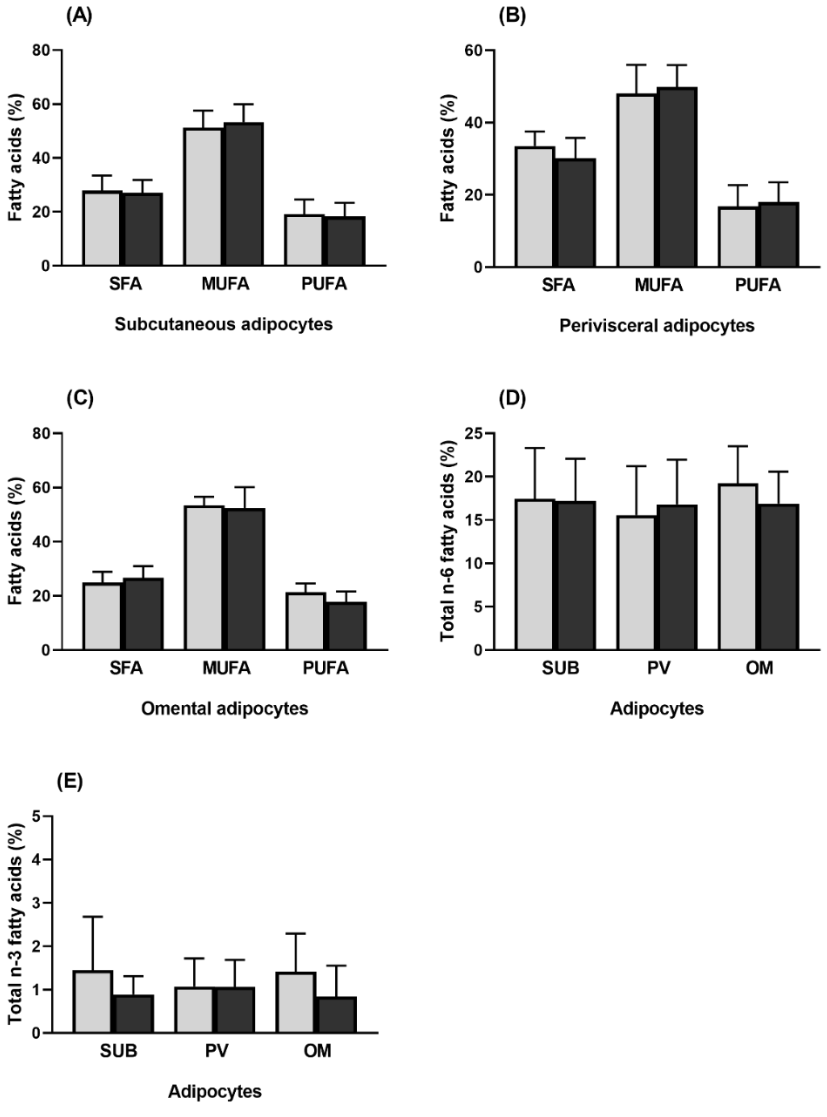

Figure S1. Fatty acids composition in subcutaneous, perivisceral and omental adipocytes (Population 2). Data were analyzed by Student t test or Mann Whitney U test as appropriate. (**A**) Fatty acids composition in SUB adipocytes in MHO and MUO subjects. (**B**) Fatty acids composition in PV adipocytes in MHO and MUO subjects. (**C**) Fatty acids composition in OM adipocytes in MHO and

MUO subjects. (**D**) Total n-6 fatty acids in the three regions of adipocytes in MHO and MUO subjects. (**E**) Total n-3 fatty acids in the three regions of adipocytes in MHO and MUO subjects. Grey bars indicate MHO and black bars indicate MUO. MHO, metabolically healthy obesity; MUFA, monounsaturated fatty acids; MUO, metabolically unhealthy obesity; OM, omental; PUFA, polyunsaturated fatty acids. PV, perivisceral; SFA, saturated fatty acids; SUB, subcutaneous.
